# Drug target, class level, and PathFX pathway information share utility for machine learning prediction of common drug-induced side effects

**DOI:** 10.3389/fdsfr.2023.1287535

**Published:** 2023-11-23

**Authors:** Han Jie Liu, Jennifer L. Wilson

**Affiliations:** Department of Bioengineering, University of California—Los Angeles, Los Angeles, CA, United States

**Keywords:** machine learning (ML), drug development, drug safety, domain knowledge analysis, drug target, protein-protein interaction (PPI) networks, drug side effect prediction

## Abstract

**Introduction:** Development of drugs often fails due to toxicity and intolerable side effects. Recent advancements in the scientific community have rendered it possible to leverage machine learning techniques to predict individual side effects with domain knowledge features (i.e., drug classification). While several factors can be used to anticipate drug effects including their targets, pathways, and drug classes, it is unclear which domain knowledge is most predictive and whether certain domain knowledge is more important than others for different side effects.

**Methods:** The goal of this project is to understand the predictive values of drug targets, drug classification (i.e., level 2 ATC codes), and protein-protein interaction networks (i.e., PathFX targets and network proteins) for machine learning prediction of 30 frequently occurring drug-induced side effects.

**Results:** We compared the prediction accuracy for individual side effects of trained models across five domain knowledge combinations and discovered that level 2 ATC codes have the highest predictive value across the domain knowledge features. Logistic regression coefficient analyses further suggest that side effects are more dependent on drug targets and drug classes, and less so on PathFX targets and network proteins.

**Discussion:** Our quantitative assessments may inform the development of safe and effective drugs by understanding the domain knowledge features underlying frequently occurring drug-induced side effects.

## 1 Introduction

The development of drugs often fails during clinical trials due to toxicity and intolerable side effects. Sun et al. (2022) analyzed clinical trial data from 2010 to 2017 and found that over 30% of drugs failed due to unmanageable toxicity. Furthermore, off-target toxicity from drugs can trigger dangerous side effects and cause clinical trial failure (Lin et al., 2019). For instance, the kinase inhibitor Sunitinib is known to trigger cardiotoxicity through its interaction with proteins outside of what the drug was intended to bind (Force and Kolaja, 2011). Currently, there are strict guidelines and protocols set in place by the United States Food and Drug Administration (FDA) to ensure drug safety and efficacy. Despite this, many drugs that are approved on the market have intolerable adverse side effects documented. Notably, propranolol hydrochloride, despite receiving approval from the FDA in 2014 for the treatment of infantile hemangiomas (Kurta et al., 2018), has been associated with sleep disturbance, agitation, and bronchial hyperreactivity (Ji et al., 2018). These findings suggest that innovation in drug development related to improved safety and efficacy could advance therapeutic development.

Multiple data-driven resources have made it possible for the scientific community to better explore the relationship between drug target (DT) associations to various side effects. Kuhn et al. (2015) generated the Side Effect Resource (SIDER) database, which documents results from public free-text data sources (i.e., literature and package inserts) using Natural Language Processing. Separately, protein-protein interaction (PPI) networks, such as PathFX, seek to understand drug-induced effects by constructing drug pathways and integrating gene-disease phenotype associations from multiple databases (Wilson et al., 2018). These drug pathways provide druggable targets and proteins downstream of targets associated with drug phenotypes. We also previously discovered that proteins downstream of druggable targets were more predictive of drug side effects as compared to DTs for severe adverse drug reactions (ADRs) listed on drugs’ labels (Wilson et al., 2022). This analysis used ADR-associated network proteins in building models and prioritized rare ADRs that have sufficient predictive power to affect the regulatory review process. DrugBank also contains domain knowledge about each drug, such as its Anatomical Therapeutic Chemical (ATC) classification and drug development group status (i.e., approved, experimental), which many have used for anticipating side effects from within-class drugs (Wishart et al., 2006).

DTs are often the starting place for predicting drug side effects. Campillos et al. (2008) demonstrated that shared drug side effect profiles were predictive of DTs. Moreover, Xie et al. (2009) explored protein-drug interaction networks of Cholesteryl Ester Transfer Protein inhibitors and identified a panel of off-target interactions that influenced side effects. LaBute et al. (2014) trained a L1-regularized LR model based on UniProt ID numbers of DTs to predict 85 side effects from SIDER grouped into 10 ADR phenotype groups and achieved a model AUC of 0.61–0.74 during a 10-fold cross-validation. However, drugs may have undocumented off-targets responsible for their effects, making DTs alone insufficient for side effect prediction.

Additional domain knowledge could improve anticipation of side effects without knowing all off-targets. Huang et al. (2011) developed a logistic regression model by integrating ADR information, DT data, PPI networks, and gene ontology term annotations to predict cardiotoxicity and achieved a performance of 0.675 in performance accuracy, the median area under the curve (AUC) of 0.771, and sensitivity of 0.632. However, this analysis was limited to predicting cardiotoxicity. They discovered that off-target proteins had more predictive power than documented on-target drug-protein interactions related to cardiotoxicity. Kim et al. (2016) leveraged ATC codes and DT information to uncover off-target tissue effects using a tissue protein-symptom matrix and predicted unintended drug side effects by off-target tissues. Further, Zhao et al. (2018) evaluated the predictive power of five domain knowledge features, namely DTs, ATC code, structure similarity, literature association of drug-protein interactions, and drug fingerprint similarity for the prediction of drug side effects with four machine learning (ML) models and achieved the highest performance when all five domain knowledge features were integrated, yielding an accuracy of 0.775. Recently, Liang et al. (2020) trained a random forest (RF) model by sampling negative cases using the random walk with restart algorithm. Furthermore, they incorporated various domain knowledge, including drug fingerprint, ATC codes, literature association of drug-protein interactions, drug structure, and DTs for the prediction of drug side effects with an RF model yielding nearly perfect performance (accuracy = 0.975). Overall, these findings suggest that incorporating DTs, PPI networks, and ATC codes for predicting drug side effects may be useful for the prediction of side effects, and leveraging more domain knowledge features may help further strengthen model performance.

Given recent successes with the integration of multiple drug data types and our previous discovery of the predictive utility of network proteins, we sought to measure the relative predictive value of DTs, drug class, and drug network proteins for the prediction of frequently occurring individual side effects in SIDER. Since ATC codes have been leveraged in building models to predict drug side effects (Liang et al., 2020), incorporating such domain knowledge in ML may provide us further insights into drug classes associated with specific organ systems that can influence frequently occurring individual side effects. A novel aspect of this project lies in the utilization of network proteins identified by PathFX. Briefly, PathFX is a pathway modeling tool for predicting drug-induced phenotypes by first identifying high-quality PPI networks around druggable targets and using functional enrichment to predict drug-associated phenotypes, while controlling for biases in network methods such as hub proteins and differential annotation in pathway phenotypes (Wilson et al., 2018). This tool is available through the command line application (Wilson et al., 2018), web server (Wilson et al., 2019), and a dockerized container including PathFX version 2 (Wilson et al., 2021). As PathFX-based predictions have been the focus of our previous work, we wanted to evaluate the utility of all network proteins instead of phenotype-specific proteins and to test our model against a broader range of side effects. By incorporating PathFX network proteins in our model, we sought to uncover certain proteins downstream of druggable targets that may influence certain side effects. The exploration of these three domain knowledge features has the potential to provide valuable insights for personalized medicine by identifying certain features that can influence drug side effects, which can assist clinicians in making informed decisions for prescribing medicine to patients. By understanding the predictive value of DTs, drug class, and drug network proteins, we can inform the therapeutic development of safer and more effective drugs to enhance patient outcomes and minimize ADRs.

## 2 Methodology

### 2.1 Extracting model inputs from data sources

#### 2.1.1 Extracting DrugBank identifiers of the 30 most common side effects from SIDER 4.1

First, we downloaded SIDER 4.1 datasets (http://sideeffects.embl.de/download/) and prioritized two of them: 1) Medical Dictionary for Regulatory Activities (MedDRA) all side effects (meddra_all_se.tsv.gz) and 2) drug names (drug_names.tsv). The MedDRA all side effects dataset contains all side effects of FDA-approved drugs documented in MedDRA. The first and last columns of the MedDRA all side effects dataset were extracted, which represent the drug ID and its associated drug side effect, respectively. Then, we mapped each drug ID to the drug name using the drug names dataset. We counted the occurrence of all side effects and noticed a drop in the number of associated drugs to 1,250 below the 30th rank. As such, we extracted the drug names associated with the 30 most common side effect counts from SIDER individually for further analysis.

Next, we standardized drug names from SIDER to a DrugBank identifier (DBID), which consists of a DB prefix and suffix of 5 numbers. Standardizing drug names to their respective DBID can increase the accuracy of mapping drugs across datasets by mitigating data loss due to differences in naming and spelling. To accomplish this, we downloaded a dataset that contains the common names and synonyms of a drug to its DBID (drugbank_vocabulary.csv). A default dictionary was generated by extracting the drug names as the key and its associated DBID as the value. The drug names from this dictionary were mapped to the drug names in SIDER 4.1 to standardize them to DBIDs.

#### 2.1.2 Running PathFX on all drugs in DrugBank 5.1.6 to obtain associated DTs and network of proteins

To extract associated DTs and PathFX network proteins for our ML input matrix, we analyzed all available drugs in DrugBank version 5.1.6 using PathFX on the Hoffman 2 cluster^1^. Briefly, PathFX generates a PPI network around DTs based on the amount and quality of evidence supporting the PPIs. Next, PathFX uses a modified Fisher’s exact test to discover biological phenotypes associated with the drug’s network (full description in Wilson et al., 2018). Importantly, PathFX can only generate a network when a drug has documented drug-binding proteins, and those proteins are connected to the PathFX interactome. Of the 13,474 drugs listed in DrugBank, PathFX generated a network file and phenotype association table for 7,012 drugs - 2,232 of which are approved on the market and 4,780 which were experimental.

#### 2.1.3 Extracting domain knowledge features to dictionaries from DrugBank 5.1.6

We sought to extract domain knowledge features associated with each DBID by storing them in dictionaries (key = DBID, value = domain knowledge feature) and subsequently appending the DBID (row) and domain knowledge features (columns) to generate the ML matrix. To assess the utility of domain knowledge for side-effect prediction, we considered 5 comparisons: 1) ATC level 2 codes only (ATC model), 2) DrugBank targets only (DT model), 3) DrugBank + PathFX targets and network proteins (DT/PathFX model), 4) DrugBank targets + ATC (DT/ATC model), and 5) DrugBank + PathFX targets and network proteins + ATC (DT/ATC/PathFX model). The level 2 ATC code consists of the first three characters of the ATC code. Since we were interested in understanding whether drug classification associated with specific organ systems influenced the prediction of common side effects, we determined that level 2 ATC codes (pharmacological and therapeutic subgroup classification information) were sufficient in providing the necessary specificity for our interests as a domain knowledge feature. There are currently 94 distinct level 2 ATC codes, each one of them indicating the system of action of the drug and its associated pharmacological and therapeutic properties. For example, C08 are calcium channel blockers that influence the cardiovascular system. We extracted both the level 2 ATC codes and DTs and generated a set dictionary with its associated DBID from DrugBank version 5.1.6. All PathFX targets and network proteins were extracted from the “merge_neighborhood_.txt” files for all 7,012 drugs using the os.walk function. Ultimately, these sets were merged using the union operator to generate the dictionaries for the five experimental conditions.

### 2.2 Matrix generation and filtering for the 30 most common side effects

For each of our five combinations of domain knowledge, we generated a ML input matrix where each row indicated a drug and the columns included a label of 1 (presence) or 0 (absence) of a domain-knowledge data type: 1) DrugBank target, 2) a PathFX target or network protein, or 3) a level 2 ATC code. We repeated this process for the top 30 side effects and created 150 data matrices in total (30 side effects x 5 combinations of predictor variables). Since SIDER 4.1 only documents side effects observed in FDA-approved drugs, we generated a subset of the matrix by excluding drugs that were not FDA-approved (i.e., experimental drugs). As adverse drug events (ADEs) are influenced by absorption, distribution, metabolism, and excretion (ADME) processes, we included genes related to these processes (CYPs, DPYD, TPMT, UGTs, and SULTs) that were documented in DrugBank.

### 2.3 Machine learning model implementation

We selected six ML models from scikit-learn capable of performing binary classification for initial evaluation and selection. Specifically, we selected the logistic regression (LR) model, Random Forest Classifier (RFC), Support Vector Machine, Decision Tree Classifier, Naive Bayesian Classifier, and K-Nearest Neighbor model on an 80/20 train-test split with random undersample of negative cases using the imblearn.under_sampling function to evaluate its accuracy in predicting dizziness, the side-effect associated with the most drugs, on DTs of FDA-approved drugs.

Next, we used the two highest-performing models from our initial evaluation and compared their performance on 30 individual side effects for model selection. We ran these models on an 80/20 train-test split with a random undersample of negative cases to balance their count with that of positive cases. This process was bootstrapped 100 times to evaluate its performance for predicting the 30 most common side effects in SIDER 4.1 using DTs only. Since the matrix contains more negative cases than positive ones, bootstrapping the negative cases can expose the model to a broader range of negative instances to improve its generalization. We compared the predictive value of both models on these side effects and selected the model that had the higher average accuracy across the 30 individual side effects.

**FIGURE 1:**
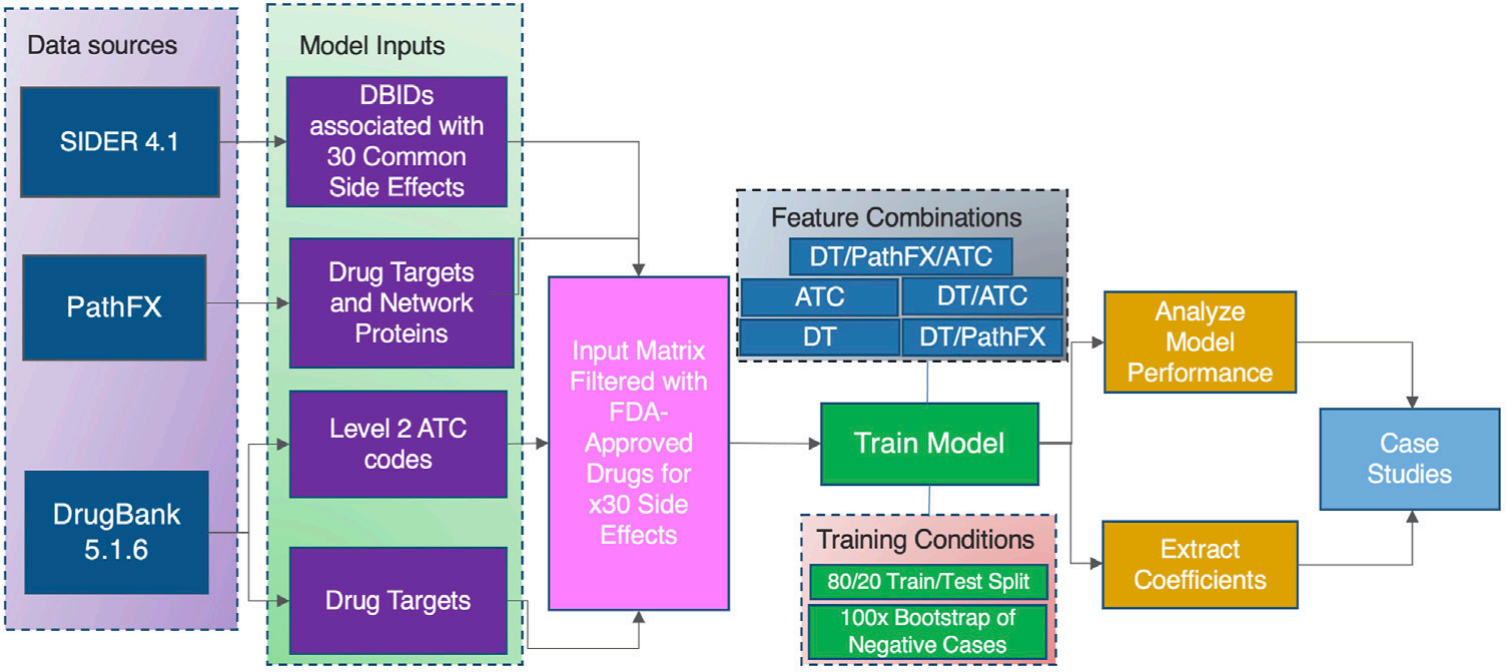
A high-level overview of our model construction and evaluation process to identify the predictive value of domain knowledge features on the 30 most frequent occurring drug side effects in SIDER.

We trained our model on five combinations of domain knowledge features on our highest performing classification model identified. Notably, we trained our model according to the training conditions listed in Figure 1 on the following groups: 1) level 2 ATC codes (ATC model), 2) DTs (DT model), 3) DTs and PathFX proteins (DT/PathFX) model, 4) DTs and ATC codes (DT/ATC), and 5) DT, PathFX proteins, and ATC (DT/PathFX/ATC) model. The model prediction accuracy across these five combinations of domain knowledge was extracted for further analyses.

### 2.4 Analysis of model performance

The initial unfiltered matrix contains both drugs approved on the market and experimental drugs, which may confound the model’s performance. To evaluate the confounding effects of experimental drugs, we compared the performance of the two models that contained 1) all drugs and 2) FDA-approved drugs using a dependent samples t-test.

We excluded all the experimental drugs in our matrix for subsequent analyses to ensure an accurate representation of these data. This is because SIDER does not document the side effects of experimental drugs, and therefore, the relationship between side effects and targets of unapproved drugs is not well established. Thus, including experimental drugs in our analyses could generate misleading results.

We performed an Analysis of Variance with Repeated Measures (ANOVA-RM) to test our hypothesis that there are between-group differences across the five combinations of domain knowledge for the prediction of individual side effects before proceeding further with subsequent analyses. Then, we performed a dependent samples t-test to assess specific between-group differences and investigated trade-offs in performance across the five combinations of domain knowledge. This approach was chosen because we did not expect one model to be uniformly more performant across all side effects. We benchmarked DTs and evaluated the change in model performance for predicting individual side effects with the addition of domain knowledge independently for the following groups: 1) DTs and PathFX proteins (DT/PathFX) model, 2) DTs and ATC codes (DT/ATC) model, and 3) DTs, ATC Codes, and PathFX proteins (DT/PathFX/ATC) model. We chose a significance level of 0.05 for all our tests.

### 2.5 Extracting LR coefficients to evaluate confounding effects and drug to side-effect associations

We extracted the LR coefficients from the trained model to understand which domain knowledge variables the model prioritized. In this project, the p variable of the LR model Eq. 1 represents the probability that the side effect of interest will occur. The p threshold of our LR model is set to 0.5, in which any value greater than 0.5 will be classified with an output label of 1 (presence of side effect). The LR model assigns a coefficient to each variable based on the outcome variable as shown in Eq. 1, where the β terms represent the coefficients and X represents the value of the predictor variable. Positive β terms suggest that an increase in the corresponding predictor variable leads to an increase in the outcome variable. Conversely, negative β terms imply that an increase in the corresponding predictor variable leads to a decrease in the outcome variable. The magnitude of the coefficient reflects the strength of the relationship between the predictor and outcome variable. These coefficients are then extracted to evaluate 1) the confounding effects of DTs unapproved in the market and 2) the validity of the suggested drug-to-variable relationship for individual side effects through case studies.

$$p=\frac{1}{1+e^{-\left(\beta_{0}+\beta_{1}X_{1}+\beta_{2}X_{2}+.\;\ldots .+\beta_{n}X_{n}\right)}}$$ (1)

### 2.6 Software and code

The data collection, processing, and model training were conducted in Python version 3.7 using Jupyter Notebook version 6.3.0. The packages deployed for this project included: 1) Pandas, Numpy, and Pickle for data processing, 2) Matplotlib and Seaborn for data visualization, 3) Imbalanced-learn to balance the binary cases, 4) Scikit-learn for modeling processed data and evaluating results, and 5) Scipy for statistical analyses.

## 3 Results

### 3.1 Data characteristics

SIDER 4.1 documented 309,848 side effects across 1,430 drugs. SIDER splits side effects based on their classification in the medical dictionary for regulatory activities (MedDRA) as either 1) preferred terms (PTs), which is a distinct medical concept for the associated side effect (i.e., nausea), or 2) lowest level terms (LLTs), which parallels how information is communicated to patients (i.e., feeling queasy). Each LLT is linked to only one PT, whereas each PT has at least one LLT. Because of this, nearly all drugs to side effect combinations may be documented multiple times. We extracted the top 30 most common side effects based on both PTs and LLTs from SIDER, with dizziness having the highest count (*n* = 2,826, including all mentions of LLTs and PTs) and musculoskeletal discomfort having the 30th-most count (*n* = 1,255) in our analysis. Below the 30th rank, the number of associated drugs dropped below 1,250, and to focus our analysis, we emphasized the top 30 drugs. After mapping to DrugBank identifiers and retaining unique drugs based on both LLTs and PTs, the side effect associated with the highest number and lowest number of unique drugs in our analysis is nausea (n = 1,207) and arthralgia (n = 588), respectively. We next matched SIDER drugs to DBIDs for integration with other data sources. Of the 1,430 drugs listed in SIDER, 1,079 of them mapped to a DBID. The percentage of drugs matched to a DBID ranged from 79.7% to 86.7% per side effect. Our original ML input matrix consisted of 7,012 drugs with documented targets or PathFX network proteins—2,232 approved drugs and 4,780 experimental, unapproved drugs. The percentage of DBIDs from SIDER that matched our ML input matrix, which consisted of DBIDs associated with a documented target or PathFX network proteins, ranged from 90.4% to 95.4% depending on the domain knowledge (some drugs did not have documented targets or PathFX networks). The sample size for each bootstrap iteration was double the number of positive DBIDs matched to the ML input matrix, as each side effect was trained on a balanced set of positive and negative DBIDs. The specific sample size ranged from 908 to 1,800 samples depending on the side effect. For instance, the bootstrap size for nausea was 1,800, as there were 900 DBIDs associated with this side effect that mapped to our ML input matrix. We curated a total of 88 level 2 ATC codes, 3,819 DTs, and 6,467 PathFX network genes with DTs which were included in our input matrix for further analyses. Importantly, the genes associated with ADME processes, such as CYPs, DPYD, TPMT, UGTs, and SULTs, were included in our input matrix if they were documented in DrugBank (PathFX uses all DrugBank information as input for network modeling).

### 3.2 Logistic regression and random forest outperform other ML models for initial side effect prediction

We first benchmarked six ML models on the most common side effect documented in SIDER: dizziness. We specifically modeled dizziness using 1) Logistic Regression (LR) model, 2) Random Forest Classifier (RFC), 3) Support Vector Machine, 4) Decision Tree Classifier, 5) Naive Bayesian Classifier, and 6) K-Nearest Neighbor model and discovered that RFC had the highest, and LR had the second highest performance as shown in Table 1. Thus, we considered these two models in subsequent additional analyses.

**TABLE 1:** Prediction performance of dizziness using FDA-approved DTs with multiple ML models.

Binary classification model	Precision		Recall		F-1 Score		Accuracy
	Negatives (*n* = 156)	Positives (*n* = 162)	Negatives (*n* = 156)	Positives (*n* = 162)	Negatives (*n* = 156)	Positives (*n* = 162)	
Logistic Regression	0.64	0.67	0.69	0.62	0.66	0.65	0.65
Random Forest	0.64	0.67	0.68	0.64	0.66	0.65	0.66
Support Vector	0.63	0.65	0.63	0.65	0.63	0.65	0.64
Decision Tree	0.61	0.67	0.72	0.55	0.66	0.60	0.63
Naive Beysian	0.73	0.57	0.29	0.90	0.42	0.70	0.60
K-Nearest Neighbors	0.56	0.69	0.82	0.39	0.67	0.50	0.60

### 3.3 Logistic regression has the highest average prediction accuracy across side effects

We analyzed the top 30 most frequent side effects in SIDER, using targets alone to predict the occurrence of the side-effect compared to non-side-effect associated drugs using both approved and experimental drugs. We further completed these prediction tasks using RFC and LC models and measured their accuracy across side effects. The LR model had a higher average accuracy (0.67) compared to the RFC (0.66) for prediction across all 30 side effects. The side-effect with the highest LR prediction accuracy was thrombocytopenia with a prediction accuracy of 0.71. The side-effect with the lowest LR prediction accuracy was infection with a prediction accuracy of 0.6.

### 3.4 Drugs targets of unapproved drugs confounded LR performance

We analyzed the confounding effects of unapproved DTs by predicting the 30 most common SIDER side effects on DTs with LR on a 100-repeat bootstrap for all drugs and approved drugs only. The model accuracy was higher when all drugs were included across all side effects compared to approved drugs only. Specifically, the mean model accuracy ranged from 0.768 to 0.833 in all drugs, and 0.612 to 0.702 in approved drugs. We hypothesized that DTs for unapproved drugs were distinct from approved drugs and influenced model performance. Of the 3,819 DTs curated; 2,543 and 2,505 DTs were associated with approved and investigational drugs, respectively. Of the unapproved drug targets (1,229/2,505, 49%) were shared with approved drugs. For this analysis, we considered the investigational targets sufficiently distinct to remove them. However, future work could include investigational drugs that targeted any targets shared with approved compounds. We further extracted the 10 most common regression coefficients exclusive to unapproved DTs and discovered that at least 5 of them were assigned a relatively negative coefficient number, suggesting that the model prioritized these targets for predicting non-side-effect-drugs. LR models for certain side effects, such as nausea, headache, and diarrhea, assigned strong negative coefficient values for all 10 most common unapproved DTs as shown in Table 2.

**TABLE 2:** Most frequent targets for experimental drugs and their regression coefficients in three example side effects: Nausea, Headache, and Diarrhea.

Target	Count	Nausea coef.	Headache coef.	Diarrhea coef.
CCNA2	66	−0.29	−0.14	−0.35
PKIA	60	−0.43	−0.42	−0.28
BACE1	56	−0.29	−0.18	−0.42
map	46	−0.40	−0.44	−0.51
MMP3	44	−0.29	−0.03	−0.31
thyA	44	−0.47	−0.32	−0.06
CTSK	44	−0.49	−0.32	−0.31
NCOA1	42	−0.26	−0.14	−0.14
CELA1	34	−0.29	−0.33	−0.55
MMP8	30	−0.49	−0.35	−0.26

### 3.5 Statistical analyses reveal three trends across different combinations of domain knowledge using LR to predict common ADEs

We repeated LR analysis for all 30 side effects using 5 different combinations of domain knowledge (see methods 2.1.4): 1) level 2 ATC codes (ATC model), 2) DTs (DT model), 3) DTs and PathFX proteins (DT/PathFX) model, 4) DTs and ATC codes (DT/ATC), and 5) DT, PathFX proteins, and ATC (DT/PathFX/ATC) model after comparing ML models and dropping unapproved drugs. We then performed an ANOVA-RM across all experiment groups to assess between-group differences across side effects. The results show significant between-group differences across all groups for the prediction of 30 individual side effects, with F-values ranging from 20.5 to 140.8, and *p*-values from 9.87E-75 to 2.42E-15 as shown in Table 3.

**TABLE 3:** ANOVA-RM LR prediction of 30 common side effects. The highest performing model value are bolded for each side effect. Each cell represents the prediction accuracy of individual common side effects from 100 bootstrapped samples trained on 1) DTs (DT model), 2) level 2 ATC codes (ATC model), 3) DTs and PathFX network proteins (DT/PathFX model), 4) DT and level 2 ATC codes (DT/ATC model), and 5) DT, PathFX network proteins, and level 2 ATC codes (DT/PathFX/ATC model). F-values indicate the ratio of variability between conditions to within conditions. *p*-values reflect the probability of obtaining the observed differences in means given the null hypothesis is true.

Side effect	DT model	ATC model	DT/PathFX model	DT/ATC model	DT/PathFX/ATC model	F-value	*p*-value
thrombocytopenia	0.71	0.70	0.67	**0.73**	0.69	85.36	3.15E-52
constipation	0.70	0.70	0.69	**0.73**	0.70	33.62	3.71E-24
somnolence	0.70	0.72	0.68	**0.73**	0.70	61.63	1.85E-40
tachycardia	0.70	0.69	0.67	**0.72**	0.68	48.88	2.08E-33
asthenia	0.69	0.72	0.69	**0.73**	0.71	90.37	1.61E-54
diarrhea	0.69	0.69	0.68	**0.72**	0.70	54.47	1.43E-36
dyspepsia	0.69	0.66	0.67	**0.71**	0.67	67.63	1.38E-43
arthralgia	0.69	0.65	0.67	**0.74**	0.69	132.52	1.01E-71
dizziness	0.68	0.68	0.65	**0.69**	0.68	38.68	2.46E-27
nausea	0.68	0.70	0.66	**0.70**	0.69	69.35	1.84E-44
rash	0.68	0.69	0.66	**0.71**	0.68	96.80	2.24E-57
abdominal pain	0.68	0.68	0.66	**0.70**	0.68	32.21	2.98E-23
headache	0.67	0.66	0.62	**0.69**	0.65	140.80	9.87E-75
dyspnoea	0.67	0.69	0.65	**0.70**	0.68	59.05	4.47E-39
anaphylactic shock	0.67	0.66	0.63	**0.69**	0.66	51.44	7.12E-35
paraesthesia	0.67	0.66	0.66	**0.70**	0.67	31.03	1.74E-22
urticaria	0.66	0.67	0.66	**0.69**	0.68	20.47	2.42E-15
body temperature increased	0.66	0.67	0.66	**0.69**	0.68	22.36	1.16E-16
dermatitis	0.66	0.68	0.68	**0.70**	0.68	71.06	2.51E-45
fatigue	0.66	0.67	0.64	**0.69**	0.67	60.49	7.54E-40
musculoskeletal discomfort	0.66	0.67	0.65	**0.71**	0.66	59.86	1.64E-39
hypersensitivity	0.64	0.66	0.61	**0.66**	0.62	110.46	3.82E-63
pain	0.64	0.66	0.63	**0.66**	0.65	33.01	9.05E-24
nervous system disorder	0.64	0.62	0.64	**0.67**	0.67	57.35	3.74E-38
vomiting	0.67	0.67	0.69	0.69	**0.70**	47.31	1.69E-32
dermatitis	0.66	0.68	0.68	0.70	**0.68**	71.06	2.51E-45
hypotension	0.66	0.70	0.68	0.69	**0.71**	45.80	1.28E-31
pruritus	0.64	0.66	0.66	0.67	**0.68**	37.13	2.24E-26
gastrointestinal disorder	0.64	0.66	0.67	0.66	**0.69**	38.19	4.96E-27
insomnia	0.64	0.69	0.67	0.68	**0.68**	47.39	1.51E-32
infection	0.61	0.69	0.63	0.65	**0.66**	125.66	3.81E-69

After identifying the presence of between-group differences across side effects, we performed several paired t-tests to identify trends among different combinations of domain knowledge. First, we compared the predictive power of level 2 ATC codes and DT for the 30 most common SIDER side effects. ATC codes were shown to be more predictive than DT for 17 drug side effects, with the largest difference in prediction accuracy between ATC codes and DTs occurring for the side effect, infection. DT was more predictive than ATC codes for only 8 side effects, with the largest difference in prediction accuracy between DTs and ATC codes being for the side effect, arthralgia. There were no significant differences in predictive power between ATC codes and DT for 5 side effects, which include constipation, abdominal pain, diarrhea, musculoskeletal discomfort, and vomiting. Overall, the predictive power was shown to be similar between level 2 ATC codes and DTs with t-test statistics ranging from −10.61 to 13.46, *p*-values from 4.19E-24 to 7.91E-01, and the average difference in accuracy being 0.01.

Next, we were interested in understanding the influence of incorporating level 2 ATC codes in addition to both DT and DT/PathFX models. The results of the LR analysis showed that the average prediction accuracy for the DT model was 0.67, while the average prediction accuracy for the DT/ATC model was 0.70. Consequently, the average prediction for the DT/PathFX model was 0.66, while the average prediction accuracy for the DT/PathFX/ATC model was 0.68. We then performed a paired t-test to assess the effect of incorporating ATC codes with DTs benchmarked with DTs on predicting the 30 most common SIDER side effects using LR at the significance level of 0.05. Incorporation of level 2 ATC codes in the DT model significantly improved model performance across all side effects, with t-test statistics ranging from −15.26 to −3.52, and *p*-values from 9.58E-28 to 6.57E-04. Incorporation of level 2 ATC codes in the DT/PathFX model significantly improved performance across all side effects, with t-test statistics ranging from −11.60 to −2.36, and *p*-values from 3.78E-20 to 2.02E-02 (Table 3).

Further, we performed a paired t-test to evaluate the predictive power of PathFX targets and network proteins on the 30 most common SIDER side effects when benchmarked with DTs alone. The addition of PathFX targets and network proteins improved LR model performance for seven side effects, which include: pruritus, vomiting, gastrointestinal disorder, dermatitis, insomnia, infection, and hypotension (Table 3).

After comparing the performance of the DT/PathFX and DT model for predicting 30 side effects, we compared the performance of the DT/ATC model to the DT/PathFX/ATC model to assess the impact of ATC codes and determine if the same side effects would be affected. Interestingly, the DT/PathFX/ATC model only improved prediction for six out of the seven side effects listed in Table 3. One unique observation is in the case of dermatitis, where the DT/PathFX model exhibited higher prediction accuracy compared to the DT model. However, the DT/ATC model surpassed the DT/PathFX/ATC model for LR prediction of dermatitis, suggesting a stronger ATC class-driven effect.

Three distinct trends for LR prediction across the 30 side effects were identified by analyzing the model accuracy of the DT, DT/PathFX, DT/ATC, and DT/PathFX/ATC models as listed below.

• Trend 1 (6 side effects): DT/PathFX model accuracy is greater than the DT model. Both these model performances improve with the addition of ATC codes, with the DT/PathFX/ATC model demonstrating the highest performance.
• Trend 2 (23 side effects): DT model accuracy is greater than the DT/PathFX model. Both these model performances improve with the addition of ATC codes, with the DT/ATC model demonstrating the highest performance.
• Trend 3 (1 side effect): DT/PathFX model accuracy is greater than the DT model. Both these models improve with the addition of ATC codes, with the DT/ATC model demonstrating the highest performance.

### 3.6 Trend 1 case study: LR prediction of gastrointestinal disorder is enhanced when level 2 ATC codes and PathFX targets and network proteins is incorporated with DTs

**FIGURE 2:**
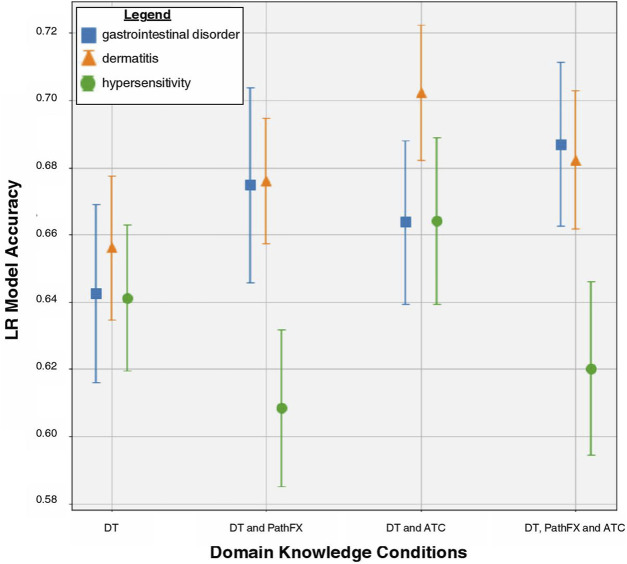
LR model accuracy for side effects of gastrointestinal disorder, dermatitis, and hypersensitivity across DT, DT/PathFx, DT/ATC, and DT/ATC/PathFX models. The square, triangle, and circle represent the mean prediction accuracy for side effects of gastrointestinal disorder, dermatitis, and hypersensitivity, respectively. Error bars represent one standard deviation of uncertainty.

Gastrointestinal disorder LR prediction accuracy increased when using all domain knowledge (accuracy = 0.69) compared with DTs only (accuracy = 0.64) and DTs with ATC codes (accuracy = 0.66) as shown in Figure 2. To better understand how LR prioritized domain knowledge, we extracted the top and bottom 30 LR coefficients for this side effect (4) and discovered that 20/30 of the largest positive coefficients were level 2 ATC codes with the largest being A10. The 30 most negative LR targets had 7/30 coefficients that were level 2 ATC codes, and 1/30 that were proteins adjacent to DTs (network downstream proteins). Overall, ATC class association and certain DTs were shown to be strong predictors of gastrointestinal disorder.

**TABLE 4:** Top and bottom 10 LR coefficients from the DT and DT/ATC/PathFX model for the side effect of Gastrointestinal Disorder. Positive coefficients suggest that the feature is positively associated with the side effect. Conversely, negative coefficients imply the feature is negatively associated with the side effect. All level 2 ATC codes adhere to the format of a letter followed by two numbers (i.e., A10). Underlined features are PathFX network proteins. The other features are DTs.

Gastrointestinal disorder							
DTs only (DT model)				Drug and PathFX targets and ATC codes (DT/ATC/PathFX model)			
Positive features	Coefficients	Negative features	Coefficients	Positive features	Coefficients	Negative features	Coefficients
XDH	1.56	NR3C1	−1.30	A10	1.97	SLC22A11	−1.17
DCK	1.28	CYP1B1	−1.03	ABCB11	1.59	folP	−1.05
HTR1B	1.20	folP	−0.97	A06	1.58	SLC10A1	−1.05
OPRK1	1.18	AR	−0.96	R03	1.38	GABRA1	−1.04
DPP4	1.14	ATP1A1	−0.95	A07	1.22	D01	−0.89
CYP3A4	1.14	CYP4A11	−0.93	J01	1.21	NR3C1	−0.80
SLC6A3	1.13	HTR2C	−0.90	L01	1.20	SLC12A3	−0.78
BCL2	1.11	ESRRG	−0.90	J05	1.13	A03	−0.76
PTGS2	1.10	CNR1	−0.90	J02	1.08	HTR6	−0.73
GNRHR	1.09	CYP2B6	−0.89	N03	1.08	pbpC	−0.68

We sought literature support for the importance of features prioritized by the LR model with all domain knowledge included. We specifically emphasized the DT, ATP binding cassette subfamily B member 11 (ABCB11), and the level 2 ATC code A10 because they had the highest coefficient values assigned in the DT/ATC/PathFX model, which had the highest performance. The evidence from the literature supports the relationship between the LR model coefficients of these variables. Chen et al. (2016) studied the effects of ingesting anti-tuberculosis drugs on Chinese individuals with the ABCB11 SNP rs2287616 and observed some adverse effects including gastrointestinal disorders, arthralgia, and pruritus. The Level 2 ATC code A10 is associated with drugs used in diabetes. An example of a drug associated with the A10 ATC code is Metformin, which is prescribed for individuals with diabetes to help control their blood sugar levels. This drug has commonly been associated with side effects of gastrointestinal disorder along with nausea, vomiting, and diarrhea, with a prevalence of 2%–63% (Siavash et al., 2017). Additionally, we were interested in understanding the role of cytochrome P450 3A4 (CYP3A4) in influencing drug-induced gastrointestinal disorder as it is prominent in phase 1 metabolism and accounts for a majority of gastrointestinal CYP activity (Thummel, 2007). Interestingly, Ketoconazole has been associated with inhibiting CYP3A4 activity in the intestinal tract, leading to a persistent inhibition of first-pass CYP3A4 metabolism and potential gastrointestinal disorder (Gibbs et al., 2000).

Further, we evaluated the relationship of negative coefficients of the DT folP and level 2 ATC code D01 on the side effect of gastrointestinal disorder. We selected folP because its coefficient values were consistently in the bottom 3 most negative values across the DT and DT/ATC/PathFX models. D01 was selected for further evaluation since it was the most negative level 2 ATC code listed in the DT/ATC/PathFX model. The evidence from the literature does not well support the relationship between the LR model coefficients of the negative variables we selected. The folP gene encodes for Dihydropteroate synthase (DHPS), an enzyme involved in the synthesis of folate in bacteria. According to Yoshida et al. (2022), inhibition of DHPS activity by Dapsone improves gastrointestinal symptoms in children with immunoglobulin A vasculitis (Yoshida et al., 2022), which contradicts the relationship that the LR model identified. The ATC code D01 is associated with Antifungals for dermatological use. Currently, the association between D01 drugs and gastrointestinal disorders is not well understood. However, the ATC code A03 is associated with drugs for functional gastrointestinal disorders, which is the 2nd most negative ATC code classified by the DT/ATC/PathFX model.

### 3.7 Trend 2 case study: DTs with ATC codes had the highest prediction accuracy for hypersensitivity

For hypersensitivity, the DT/ATC model had the highest prediction accuracy (accuracy = 0.66, Table 3). The DT/ATC/PathFX model had lower prediction accuracy than DT alone (accuracy = 0.62 compared to accuracy = 0.64) as shown in Figure 2. To better understand this trend, we extracted the top and bottom 30 LR coefficients for this side effect (Table 5) and counted the number of features that weren’t DTs in the 1) DT/ATC/PathFX and 2) DT/ATC models. We discovered that 15/30 of the largest positive variables and 3/30 of the negative variables were level 2 ATC codes in the DT/ATC/PathFX model. However, in the DT/ATC model, only 14/30 of the largest positive coefficients and 2/30 of the most negative coefficients were level 2 ATC codes. Consistent with gastrointestinal disorder, these findings suggest that ATC class association and certain DTs are strong predictors of hypersensitivity.

**TABLE 5:** Top and bottom 10 LR coefficients from the DT, DT/ATC, and DT/ATC/PathFX model for the side effect of hypersensitivity. Positive coefficients suggest that the feature is positively associated with the side effect. Conversely, negative coefficients imply the feature is negatively associated with the side effect. All level 2 ATC codes adhere to the format of a letter followed by two numbers (i.e., A10). Underlined features are PathFX network proteins. The other features are DTs.

Hypersensitivity											
DTs only (DT model)				DTs and ATC codes (DT/ATC model)				Drug and PathFX targets and ATC codes (DT/ATC/PathFX model)			
Positive targets	Coef.	Negative targets	Coef.	Positive targets	Coef.	Negative targets	Coef.	Positive targets	Coef.	Negative targets	Coef.
DNMT1	1.16	CYP3A43	−1.19	V08	1.53	CYP3A43	−1.09	DPP4	1.30	folP	−1.32
DCK	1.15	SLC16A10	−1.17	HTR1D	1.37	ADRA2C	−1.09	J05	1.22	CYP3A43	−0.93
rpsI	1.01	CYP2B6	−1.15	G01	1.26	TEK	−0.96	A02	1.22	AR	−0.90
CHRNA3	1.01	IFNAR2	−0.96	ADORA2A	1.26	M09	−0.89	MPO	1.20	HTR1E	−0.87
MTOR	1.00	ABCC10	−0.92	A04	1.12	TNF	−0.87	M03	1.18	ampC	−0.83
UGT1A9	0.98	IDH1	−0.83	DNMT1	1.11	ABCC10	−0.82	V03	1.12	A12	−0.82
GNRHR	0.97	ADRA2C	−0.82	XDH	1.09	ALK	−0.81	L01	1.11	GNRHR2	−0.80
ADRB2	0.97	NTRK1	−0.81	TSPO	1.07	CACNA1G	−0.77	J02	1.08	KCND3	−0.79
HTR3A	0.92	ADRA1D	−0.80	ABCB11	1.07	HSD3B1	−0.75	J01	1.06	H02	−0.78
PGD	0.91	SULT2A1	−0.80	J01	1.06	ATP1A1	−0.73	J04	1.01	HTR6	−0.77

We again sought literature evidence to support features that were prioritized by the LR model. Specifically, we selected the DT prostaglandin D2 (PGD), which was the 10th highest feature in the DT model, and ATC code J01, which was the 10th highest feature in the DT/ATC model, for further investigation. While limited studies have documented the direct effect of drug-induced hypersensitivity from PGD interactions, studies have shown that the PGD metabolite levels in urine are associated with the severity of hypersensitive reactions to ingested foods (Maeda et al., 2017). The ATC code J01 is associated with antibacterials for systemic use. Currently, there are drugs within the J01 ATC category that have been associated with hypersensitivity reactions, including Penicillins (Weiss and Adkinson, 1988), Cephalosporins (Moreno et al., 2008), and Sulfonamides (Slatore and Tilles, 2004). While we expected classical hypersensitivity HLA genes to be associated with drug side effects, our models surprisingly did not prioritize them as our analysis was limited to DTs documented in DrugBank or network proteins with high-quality associations to DTs. Two drugs from DrugBank have at least one HLA gene (HLA-A, HLA-DQB1, and HLA-DQA2) as their targets (insulin pork, coccidioides, and immitis, spherule), but these drugs were not associated with side effects in SIDER, so they were skipped. Interestingly, 39 HLA genes were identified in the PathFX network with 20 drugs having at least one association with those genes. Out of these 20 drugs, only three of them (Copper, Dasatinib, and Ponatinib) were documented in SIDER with Dasatinib being the only drug associated with hypersensitivity.

We subsequently evaluated the influence of DT ATP1A1 (ATPase Na+/K+ transporting subunit alpha-1) which was the 10th lowest feature in the DT/ATC model, and ATC code H02, which was the 9th lowest feature in the DT/ATC/PathFX model, on the side effect of hypersensitivity. Since ATP1A1 is involved in ion transport, it is not specifically associated with modulating hypersensitivity reactions to drugs; however, our model predicts that this protein could have a role in the hypersensitivity reaction. To our knowledge, there is currently no known association between ATP1A1 and drug-induced hypersensitivity reactions. The ATC code H02 is associated with corticosteroids for systemic use. There are several drugs within the H02 ATC category that have been used to treat hypersensitivity, including Methylprednisolone (Ocejo and Correa, 2019) and Dexamethasone (Johnson et al., 2018).

### 3.8 Trend 3 case study: LR prediction of dermatitis is increased when level 2 ATC codes are incorporated with DTs

Dermatitis LR prediction accuracy is increased when level 2 ATC codes are incorporated with DTs. Interestingly, both PathFX proteins and ATC codes improve LR performance (accuracy = 0.68) compared to DTs alone (accuracy = 0.66), but do not improve accuracy as much as level 2 ATC codes and DTs (accuracy = 0.70) as shown in Figure 2. To better understand this trend, we extracted the top and bottom 30 LR coefficients from the DT, DT/ATC, and DT/ATC/PathFX models for this side effect (Table 6) and counted the number of non-DT features. We discovered that 21/30 of the largest positive variables and 6/30 of the negative variables were level 2 ATC codes when both PathFX proteins and ATC level 2 codes were included. However, when PathFX network proteins were eliminated from the LR model, only 17/30 of the largest positive coefficients and 6/30 of the most negative coefficients were level 2 ATC codes. Given its high absolute coefficient values, our findings suggest that ATC class association is more associated with dermatitis than individual DTs and network proteins.

**TABLE 6:** Top and bottom 10 LR coefficients from the DT, DT/ATC, DT/ATC/PathFX model for the side effect of dermatitis. Positive coefficients suggest that the feature is positively associated with the side effect. Conversely, negative coefficients imply the feature is negatively associated with the side effect. All level 2 ATC codes adhere to the format of a letter followed by two numbers (i.e., A10). Underlined features are PathFX network proteins. The other features are DTs.

Dermatitis											
DTs only (DT model)				DTs and ATC codes (DT/ATC model)				Drug and PathFX targets and ATC codes (DT/ATC/PathFX model)			
Positive targets	Coef.	Negative targets	Coef.	Positive targets	Coef.	Negative targets	Coef.	Positive targets	Coef.	Negative targets	Coef.
AGTR1	1.38	folP	−1.07	D07	2.24	R06	−1.12	D07	2.38	folP	−1.30
DCK	1.35	SLC47A2	−1.01	GNRHR	1.47	SLC16A10	−1.11	N03	1.81	V04	−1.09
ORM1	1.32	CFTR	−0.95	N04	1.44	SLC10A1	−1.07	N02	1.56	SLC22A11	−0.98
ABCC4	1.14	ABCG2	−0.95	C09	1.43	folP	−1.04	M03	1.52	SLC18A2	−0.95
TSPO	1.13	PPARA	−0.92	A04	1.40	ABCC10	−1.00	B01	1.38	CYP3A43	−0.94
HTR3A	1.12	SLC16A10	−0.91	J02	1.38	TNF	−0.94	C09	1.32	S02	−0.92
UL30	1.09	SLC18A2	−0.90	C03	1.38	V04	−0.92	G02	1.23	R06	−0.91
MPO	1.09	PGR	−0.89	N03	1.35	CYP2B6	−0.90	L02	1.21	JAK2	−0.87
FDPS	1.07	SLC10A1	−0.89	G04	1.26	SLC18A2	−0.88	HTR2B	1.16	FXYD2	−0.86
PDE3A	1.03	IFNAR2	−0.89	A08	1.25	PPARA	−0.88	J05	1.15	L03	−0.86

We sought literature support for the DT, Gonadotropin-releasing hormone receptor (GNRHR), and the level 2 ATC code D07, both of which had positive coefficients for predicting the side effect of dermatitis. We selected GNRHR as the DT of interest because it had the highest positive coefficient value amongst all targets in the DT/ATC model. Further, we selected D07 because it was assigned the highest coefficient across all level ATC codes for predicting dermatitis. While there are currently limited studies that demonstrate the relationship between GNRHR and drugs on the side effect of dermatitis, Han et al. (2023) recently administered the GnRH antagonist Relugolix which revealed lichenoid dermatitis with eosinophils 9 weeks post-treatment. Relugolix has been demonstrated to lower testosterone levels fast (Shore et al., 2020). This effect may increase the risk of developing dermatitis, as previous studies show that male atopic dermatitis patients have lower testosterone levels when compared to controls (Gratton et al., 2022). The ATC code D07 is associated with Corticosteroids for dermatological preparations. This class of drugs, including Hydrocortisone (Sears et al., 1997), Betamethasone (Jensen et al., 2009), and Clobetasol (Alam et al., 2013), have been used for dermatitis treatment. However, contact sensitivity to such drugs could lead to adverse effects, such as stasis dermatitis, perineal dermatitis, and chronic actinic dermatitis (Coondoo et al., 2014).

We further investigated the DT, folP, and level 2 ATC code, R06, both of which had negative coefficients for the prediction of dermatitis. We selected folP because its coefficient values were consistently in the bottom 3 most negative values across the DT/ATC and DT/ATC/PathFX models. R06 had the largest negative coefficient in the DT/ATC model. Dapsone, an FDA-approved for dermatitis, competitively inhibits the action of DHPS to reduce inflammation associated with dermatological conditions (Kurien et al., 2022). The ATC code R06 is associated with Antihistamines. Currently, there are several antihistamines that have been found to be effective in improving dermatitis symptoms, including Cetirizine (Hannuksela et al., 1993), Loratadine (Herman and Vender, 2003), and Fexofenadine (Kawashima et al., 2003).

## 4 Discussion

Side effects in FDA-approved drugs continue to be a major concern despite the strict guidelines and protocols in place during the drug development and approval process. These side effects can significantly impact the quality of life for its users. Recent advancements in the scientific community have sought to address these issues through the development of various tools and resources such as the PathFX algorithm, SIDER, and DrugBank databases. Specifically, the PathFX algorithm identifies potential connections between drugs, targets, and downstream proteins associated with a phenotype. SIDER documents drug side effects from public free-text data sources (i.e., literature and package inserts) using Natural Language Processing. The DrugBank database assigns a standardized ID to all drugs and provides extensive information about each one of them, such as its associated ATC code, DT, and description. These resources provide crucial information in enhancing our understanding of the relationships between drugs and side effects, thereby facilitating future developments of safe and effective drugs. We, and others, have used this domain knowledge to better predict DTs with varying success. Previously we had discovered that proteins downstream of druggable targets that were associated with severe, adverse reactions, were predictive of drug outcomes (Wilson et al., 2022). We were eager to understand whether these findings applied to cases across milder, and more frequent side effects.

This project analyzed the predictive value of three types of domain knowledge—DTs, PathFX network proteins, and ATC codes - for the prediction of the 30 most common side effects from SIDER. We used the DT model as a benchmark to evaluate the predictive value of three domain knowledge combinations 1) DT/PathFX 2) DT/ATC, and 3) DT/PathFX/ATC. Our results showed the following key observations based on the three trends identified: 1) incorporation of PathFX targets and network proteins resulted in improved prediction for side effects for 7 out of 30 side effects, 2) level 2 ATC codes enhanced LR model performance for prediction of all 30 side effects, and 3) despite the DT model performing worse than the DT/PathFX model, the DT/PathFX/ATC model did not substantially improve model performance compared to the DT/ATC model for LR prediction of dermatitis. Overall, these observations suggest the following: 1) pathway information and PPIs can be useful for the prediction of certain side effects, 2) drug classification information positively impacted the accuracy of side effect predictions, and 3) incorporation of both PathFX targets and level 2 ATC codes may not significantly influence the prediction accuracy compared to level 2 ATC codes alone for prediction of certain side effects. We further extracted the top and bottom 30 LR coefficients of three individual side effects from each identified trend to gain a better understanding of the features that our models prioritized. The LR model prioritized both DTs and level 2 ATC codes, further implying that drug-target interactions and drug classification may be informative in understanding side effects. Since drugs within the same class share common characteristics in terms of their mechanism of action, chemical structure, or intended therapeutic use, this may suggest that therapeutic targeting of certain organ systems is sufficient for causing side effects, irrespective of distinct DTs. Further, drugs with shared ATC codes often share DTs, and these shared properties between level 2 ATC codes and DTs could potentially explain the similarities in their predictive power. However, the LR model did not often prioritize PathFX targets and network proteins, which suggests that pathway information and protein interactions may be relatively less influential in predicting individual side effects. Indeed, in our initial PathFX analysis, we discovered that downstream proteins were more predictive for certain disease indications, and it's reasonable that frequent side effects may not be well-explained by additional downstream proteins. This is further supported by Huang et al. (2011), who noted that off-target proteins were more predictive of drug-induced side effects, though this was limited to cardiotoxicity.

There are some limitations to our method. First, our trained models can be potentially better fine-tuned to achieve more optimal performance. While we were primarily interested in investigating how model performance changes across different combinations of domain knowledge, fine-tuning the model to improve the performance of our model may lead to a more accurate representation of coefficient assignments in the LR model. The suboptimal accuracy of our model may have led to our model inaccurately assigning negative coefficients. For example, in the hypersensitivity case study, there was no known association between ATP1A1 and drug-induced hypersensitivity reactions to date. As such, future work will explore training models that include all domain knowledge and employ a feature selection approach to optimize model performance. By doing so, we aim to better understand the predictive value of domain knowledge features for each individual side effect. Second, our trained LR model only considers three areas of domain knowledge (DTs, PathFX targets, network proteins, and level 2 ATC codes), which may limit its performance potential. Further, compared to our previous discovery, we did not restrict PathFX proteins to side-effect-associated proteins. Third, our analyses were limited to only the top 30 most common side effects. Future work can expand the scope to explore a broader range of side effects. Fourth, we limited our analyses to drug targets of approved drugs and excluded those that were unique to unapproved drugs. As such, future work could include investigational drugs with comparable or related targets. Fifth, although various research groups have achieved success in leveraging level 4 and 5 ATC codes in predicting drug side effects (Cami et al., 2011; Zhao et al., 2018; Galeano and Paccanaro, 2022), we opted to use level 2 ATC codes. Given that we were primarily interested in learning whether classification associated with specific organ systems influenced the prediction of common side effects, we determined that level 2 ATC codes provided the necessary specificity for our interests. As such, future work can explore whether the predictive value of ATC codes changes with more specific terms. Lastly, we discovered literature evidence for only a handful of LR coefficient associations; they supported the features prioritized by our models, but investigations are needed to further validate the coefficient associations identified by the LR model and affirm the importance of model-prioritized features.

Previous studies have explored the use of ATC codes and DT information to predict general or specific drug-induced side effects. However, these studies did not specifically focus on common drug-induced side effects. Kim et al. (2016) analyzed the utility of drug off-targets in predicting side effects by identifying relationships in the tissue protein-symptom matrix. While this study leveraged DT information to uncover off-target tissue effects, it does not directly address the predictive power of DT information for the prediction of individual side effects. Further, Zhao et al. (2018) evaluated the predictive power of five domain knowledge features, namely DTs, ATC code, structure similarity, literature association of drug-protein interactions, and drug fingerprint similarity for the prediction of drug side effects with four ML models. The RFC model achieved the highest performance when all five domain knowledge features were integrated, yielding an accuracy of 0.775. Despite achieving a higher prediction accuracy through the integration of multiple domain knowledge features, Zhao et al. (2018) did not specifically aim to assess its utility in predicting individual side effects. Lastly, Huang et al. (2011) trained an LR model that combined DT data, PPI networks, and gene ontology annotations for the prediction of side effects of experimental drugs and achieved an accuracy of 0.675 for the prediction of cardiotoxicity. However, the study’s claim of predicting cardiotoxicity with experimental drugs may be limited. First, Huang et al. (2011) used drugs from SIDER, which primarily documents the side effects of FDA-approved drugs. Second, their study depended on molecular docking information, and they did not incorporate protein structural information in their model. Last, they only trained their model to predict one type of side effect: cardiotoxicity.

Compared to other cited examples, we are generally on par with or exceed other approaches, with the exception of Liang et al. (2020), who trained RF models that yielded nearly perfect performance (accuracy = 0.975). The moderately high but consistent performances across approaches with distinct domain knowledge underscore the difficulty in predicting drug side effects generally. Additionally, in our comparison of the value of each component of domain knowledge, we discovered that none of DT, ATC, or PathFX had a drastic improvement in performance with relatively minor changes in AUC values. This result suggests some redundancy in domain area knowledge. Of the five domain knowledge features in Zhao et al. (2018), the exclusion of DTs and ATC codes had the least impact on the overall model. This suggests that the inclusion of additional domain knowledge, such as drug similarity, literature association of drug-protein interactions, and protein structural information, can potentially improve the performance of our model. Further, Huang et al. (2011) discovered that additional off-target information was of high predictive value for predicting side effects. Given the various approaches to predicting drug-induced side effects, future work could emphasize discovering negative examples that are distant from positive cases based on drug features, as exemplified in Liang et al. (2020). Additionally, the inclusion of predicted drug-binding protein targets could be a potential avenue for exploration as it has exhibited relatively high predictive value, as shown in Huang et al. (2011). In ongoing work, we are actively exploring the utility of predicted drug-binding proteins for improving PathFX predictions, but that work was outside the scope of this initial analysis.

In this study, we are interested in identifying associations between common drug-induced side effects and domain knowledge features to inform the development of novel therapies with known or tolerable side effects. Although previous studies have leveraged DTs, ATC codes, and PPI networks for the prediction of side effects, limited studies have assessed the predictive value of ATC codes, DT information, and PathFX targets and network proteins for predicting individual side effects. Consistent with our hypothesis, this study showed that LR model performance changes with the inclusion of domain knowledge for prediction across 30 individual side effects. LR coefficient analyses further suggest that side effects may be more heavily influenced by DT and classification information. For us and others, these findings highlight the importance of considering organ-system information in engineering pathways and considering potential off-targets that could be connected to side-effect outcomes. Bridging these gaps could advance network methods to have better predictive utility and generally enhance our ability to anticipate common drug-induced side effects and inform future drug development.

## Data Availability

The datasets presented in this study can be found in online repositories. The names of the repository/repositories and accession number(s) can be found below: https://github.com/jenwilson521/ML_Atc_DT_PFX.

## Footnotes

1
https://www.hoffman2.idre.ucla.edu/
